# Does Internet Entertainment Reduce the Cognitive Ability of Children? Evidence from the China Education Panel Survey

**DOI:** 10.3390/bs12100364

**Published:** 2022-09-27

**Authors:** Wenxin Hu, Yufei Mao, Kevin Huang, Yanqi Sun

**Affiliations:** 1 School of Economics and Management, Beijing Institute of Petrochemical Technology, Beijing 102617, China; 2 School of Labor Economics, Capital University of Economics and Business, Beijing 100070, China; 3 School of Accounting, Economics and Finance, University of Wollongong, Sydney, NSW 2522, Australia

**Keywords:** internet entertainment, children, cognitive ability, family environment, influencing mechanism

## Abstract

Internet technology has been assimilated into children’s educational system on an in-depth level. In particular, the number of children who use the internet for entertainment has been rapidly increasing. However, there has been a debate as to whether internet entertainment can have a detrimental impact on children’s cognitive ability. This paper investigates the effect of internet entertainment on the cognitive ability of children in the Chinese context. The results show no evidence of associations between internet entertainment and children’s cognitive ability. However, the additional analysis provides preliminary evidence suggesting that internet entertainment can be beneficial to children who use it for entertainment only on weekends but detrimental for those who spend leisure time online daily. In addition, the findings are robust in a variety of sensitivity tests. We also examine whether the effects of internet entertainment on children’s cognitive ability in different family environments are heterogeneous. The findings suggest that parents’ internet habits, parents’ internet supervision, parental relationship, family education and living area play a moderating role in the relationship between internet entertainment and children’s cognitive ability. This study offers useful insights into the current global debate on the nexus between internet entertainment and children’s cognitive ability and also provides suggestions for parents, children, regulators and policymakers.

## 1. Introduction

As an essential part of modern education, internet technology has been gradually incorporated into the children’s education system by governments and education institutions worldwide [1]. However, children’s internet entertainment is controversial and has shown both benefits and costs [2]. Internet entertainment refers to individuals using an internet platform for online games, online videos, internet news, online music and other entertainment activities, and it is one of the main internet applications at present. On the one hand, information searching, access to online literature and online education derived from internet technology can help children to extend their knowledge and alleviate the information lag caused by remoteness and poverty [3]. On the other hand, the internet is very attractive to children. Excessive internet usage may affect children’s learning and even cause significant harm to their physical and mental health. Internet addiction can be problematic, and this is a public consensus [4]. In 2019, an article entitled “Where Games Will Take Left-behind Children” sparked a heated debate about children’s internet usage. The report argued that the neglect of parental care and a sense of meaninglessness in life aggravate the negative impacts of online games on left-behind children in rural areas, seriously hindering children’s health and development [5]. With the rapidly increasing number of internet users in China, many citizens start to use the internet at a much younger age than previously. According to the 2015 Chinese Youth Online Behavior Report released by the China Internet Network Information Center (CNNIC), the number of online children in China had reached 287 million, with an internet penetration rate of 85.3%, far higher than the overall national level of internet usage, 50.3%. Nearly 70% of online gamers were students, far exceeding the proportion of other netizens, which was 13.1% (CNNIC, 2016). Due to the heavy learning tasks of minor children, their bodies and minds are not yet mature. As they have poor self-control, minor children easily become addicted to the internet and are unable to control the time they spent on the internet. Thus, developing appropriate rules, such as those that guide children to correctly use the internet for entertainment and prevent internet addiction, has become a focus for the education sector and the community.

Cognitive ability is an important indicator for measuring children’s development, and it significantly impacts their future socioeconomic outcomes and behaviors [6]. Cognitive ability refers to the ability to use the human brain to process, store and extract information and mainly involves abstract thinking, logical deduction and memory ability [7]. Previous studies have explored the influencing factors of children’s cognitive ability from the perspectives of early health, parental participation, preschool education and family economy [8,9]. The latest, relevant literature also paid attention to the relationship between internet use and children’s subjective well-being and believed that only when internet use is expressed as problematic does it have a negative impact on subjective well-being. On the contrary, when there is no problem expressed with the use of the internet, the effect is positive [10]. With the popularization of computer education in schools and internet usage at home, scholars have begun to pay attention to the influence of the internet on children’s development. The findings of prior studies are not conclusive.

Some studies had a positive attitude and found that internet entertainment is positively related to children’s math and reading scores, science and technology and academic interests [11]. Other studies analyzed the impact of home internet access on the cognitive ability gap between urban and rural children and found that students with an urban registered residence perform better in internet use because their cognitive ability is higher, and they are more inclined to use the internet as a means of searching for information and learning [12].The internet conveniently facilitates access to information and the personalization of teaching, which is beneficial for children’s horizons, logical operation and creative thinking, especially for children in remote and poor areas.

However other studies underlined the opposite findings. They reported that the use of the internet does not lead to higher children’s cognitive ability and increased academic performance. Furthermore, excessive internet usage harms children’s cognitive ability, increases the chances of vision loss and the risk of obesity and reduces sleep quality and social participation. Excessive internet usage leads to internet addiction, violent behavior, psychological depression and other potential hazards [2]. Other studies focused on the impact of new media, such as the mobile internet, on left-behind children. They believed that the mobile internet has a variety of effects on the cognition and behavior of left-behind children. Due to the lack of correct guidance, the results showed that the disadvantages outweigh the advantages [13]. Considering that the internet is a multi-functional technology, assessing its benefits and costs depends largely on the actual usage time [14]. Children under the age of 18 are more likely to use the internet for entertainment purposes. Their preferences for using the internet at different times, such as on weekdays or weekends, may have other effects on cognitive ability.

Based on the new human capital theory, learning cognitive theory and time substitution theory, this paper used 10,934 samples of CEPS data to explore the effect and mechanism of internet entertainment on children’s cognitive ability. It was found that internet entertainment has no significant impact on children’s cognitive ability, but additional analysis provided preliminary evidence suggesting that internet entertainment can be beneficial to children who use it for entertainment only on weekends but detrimental for those who spend leisure time online daily, and the impacts of internet entertainment are heterogeneous in different family environments.

The contributions of this paper are the following aspects: First, it enriches the relevant research on the relationship between internet usage and children’s development. At present, many scholars at home and abroad have conducted rich discussions on children’s internet use and urban–rural differences and their relationship with academic performance. Most of the literature has focused on children’s overall internet use, but fewer pieces of research were concerned specifically with internet entertainment. Moreover, the concepts of academic performance and cognitive ability are not same, and the existing conclusions cannot be directly applied. Different from the research perspective in previous studies, this paper focuses on the impact of internet entertainment on children’s cognitive ability in combination with the actual online situation of children in middle school and puts forward hypotheses based on existing theories, which enriches the research in related fields. Second, existing research on children’s internet entertainment has often used qualitative research methods, such as literature research, case analysis and in-depth interviews, but few studies explored quantitative methods. Based on 10,934 samples of children aged from 13 to 15 in junior middle school, this paper builds an econometric model and empirically tests the influence of internet entertainment on children’s cognitive ability and carries out quantile regression and a robustness test in a panel data model. Third, this paper adds a discussion on the heterogeneity of family environment. Family is the sum of subjective and objective conditions in which children live and which children rely on for growth and development. When discussing the relationship between internet entertainment and children’s cognitive ability, it is of great practical significance to consider the role of family environment. In previous studies, children were often regarded as a homogeneous group. Because of the differences in their home network environment, educational resources and living areas, the effects of internet entertainment on children’s cognitive abilities may be different. Therefore, it is necessary to distinguish the heterogeneity of the family environment. In fact, for children in different family environments, the influencing factors of their cognitive abilities are heterogeneous. In view of this, this paper conducts sample analysis according to the family network environment, educational resources and residential areas and investigates the heterogeneity of influencing factors of cognitive abilities in different family environments. Fourth, this paper also discusses the influencing mechanism between internet entertainment and children’s cognitive abilities and explains it from the perspectives of the cognitive effort, continuous learning and life attitude of internet entertainment.

## 2. Literature Review and Research Hypothesis

### 2.1. Internet Entertainment and Children’s Cognitive Ability

New human capital theory regards the development and formation of cognitive ability as a dynamic process and posits that cognitive ability has high plasticity in the stage of childhood [15]. Therefore, discovering how to promote the development of children’s cognitive ability has represented a hot topic for researchers and practitioners. Explanations of how internet entertainment affects children’s cognitive ability are different from distinctive theoretical perspectives [16]. Psychological theories, such as cognitive learning theory and arousal theory, posit that internet entertainment may generate a negative effect on the development of children’s cognitive ability [17]. This is because children can easily capture information via the internet. Therefore, they are no longer willing to invest cognitive efforts in addressing complex problems. In addition, internet entertainment, such as online games, rewards children in a more timely manner compared with learning activities in the real world. Children use internet entertainment to satisfy their sense of achievement, thus, reducing their cognitive effort in real life. Moreover, children’s excessive internet entertainment may distract them from their learning and cause emotional disturbance [18]. This is because the information received by children through internet entertainment has high variety and velocity. Characteristics such as high arousal and rapid presentation reduce children’s attention and continuous learning engagement, interfere with normal emotional stability and cause impulsive behavior. At the same time, children playing online games may also be exposed to some undesirable content, which harms life attitudes and social behavior [19]. Economic theories mainly interpret the influence of internet entertainment from the perspective of time allocation. From the point of time distribution, everyone’s time is limited. Internet entertainment has a substitution effect in relation to children’s learning and extra-curricular activities. If the time used to enjoy internet entertainment increases, it could reduce time spent on other extra-curricular activities, and excessive internet entertainment reduces children’s study time.

Although it is indisputable that excessive internet entertainment brings about many adverse effects on children’s development, the impact of internet entertainment on cognitive ability is not without a single benefit. Empirical studies have shown that internet entertainment, such as video games, can improve children’s visual sense of space and problem-solving skills and has a positive impact on their math performance and teamwork capabilities [3,20]. In addition, there are some entertainment applications, such as puzzle games, online videos and online music, which also help children to adjust their life–study balance, relieve their stress and relax their moods [21]. In addition, children can be exposed to new technologies at an early age through internet entertainment, which promotes their computer skills and, thus, improves their work performance in adulthood [22]. From this point of view, internet entertainment does not always harm children’s cognitive ability. Under certain conditions, it also becomes an effective tool to promote children’s active thinking and capability development. Moderate internet entertainment, which plays a minor substitution effect role in relation to everyday learning and extra-curricular activities, can help to relax mood and relieve learning pressure. However, excessive internet entertainment occupies significant time for children which may have otherwise been spent on conducting readings, physical activities and social interaction, which may further lead to children’s dependence and addiction to the internet [20]. In reality, some children have good internet habits. They often just use the internet for entertainment purposes on the weekend after meeting their learning goals. However, some other children have very poor self-control. They usually stay on the internet for entertainment on all days of the week. The influences of surfing the internet at different times on cognitive ability can be different. Given the above analysis, this paper proposes the following hypothesis:

**Hypothesis** **1** **(H1).** *Using the internet for entertainment on weekends is beneficial to children’s cognitive ability. However, using the internet on weekdays and weekends for entertainment has a negative effect on their cognitive ability.*

### 2.2. Heterogeneous Effects of Internet Entertainment in Different Family Environments

The family environment is a key place for children’s growth [23]. A large number of studies have shown that elements of a family environment, including family internet environment, family education resources, the relationship between the parents and family economic conditions, not only have an effect on children’s academic performance, body health and mental development, but can also affect children’s internet entertainment behaviors [24].

In terms of the family internet environment, first of all, whether there is internet at home directly impacts children’s internet entertainment behaviors. Having internet at home reduces the cost of children’s internet access, enables children to master information technology skills earlier than those in families without internet access and makes it more convenient to surf the internet at home [7]. However, children with poor self-control are more likely to spend too much time on internet entertainment rather than on other more valuable extra-curricular activities, which has a more apparent negative impact on cognitive ability [25]. Secondly, parents’ internet usage habits have a demonstrative effect on children’s online behavior. Compared with parents who do not surf the Internet regularly, parents with good internet habits can better filter the junk information on the internet. They can correctly guide children to choose more beneficial internet content, thus, reducing the negative impact of internet entertainment on children. In addition, parents can strictly supervise their children’s access to the internet and effectively urge them to arrange their entertainment time by formulating internet rules, which is conducive to the formation of children’s good internet usage habits. Children with less strict parental supervision are more likely to indulge in excessive internet entertainment, which further increases the negative impact on cognitive ability [23,26].

From the perspective of other family characteristics, in terms of family education, families with parents with higher educational backgrounds or cultural capital pay more attention to the early investment in education and the overall improvement of children’s abilities [27]. They are more likely to master network information technology skills and are capable of guiding children to use the internet appropriately. Children are also influenced by their parents to raise their educational expectations. Therefore, children with better family education resources have higher self-control ability when accessing the internet, and the negative influence of internet entertainment is relatively low [28]. In terms of family relations, families with good parent-related relationships provide children with a harmonious growing environment. A family environment in which parents always communicate with children and care about their daily life is more conducive to controlling children’s internet entertainment time. However, in families where children have poor relationships with their parents, children often lack the feeling of warmth and care and are more likely to seek compensation from internet entertainment, which is more likely to lead to the destructive behaviors caused by internet addiction [29].

**Hypothesis** **2** **(H2).** *The effects of internet entertainment on children’s cognitive ability in different family environments are heterogeneous. The positive effect of internet entertainment is more significant for children in families where the parents have good internet habits, higher education and a harmonious relationship with their children.*

## 3. Methodology

### 3.1. Sample and Data Sources

The data used in this paper came from the 2013–2014 China Education Panel Survey (CEPS); the changes of children’s cognitive abilities are very slow, and, thus, the level of children’s cognitive abilities remains stable for a long time. The survey was designed to investigate children’s cognitive ability and internet entertainment for each of the 31 Chinese provinces, and all the participants completed the survey in the same period. The research samples were mainly children aged from 13 to 15 in junior middle school. The total number of questionnaires was 19,487, after eliminating the samples with missing key variables, the final number of samples was 10,934.

Explained variables: cognitive ability. The CEPS project used the internationally standardized test to quantify the children’s cognitive ability in three dimensions: language, graphics and space, computation and logic. In the test, students were required to complete all questions in class, and the time was strictly controlled, effectively ensuring the comparability of the results at the national level. This paper selected the cognitive ability standardized score based on the 3PL model to measure children’s cognitive ability. In addition, in the robustness test, students’ self-assessment scores and standardized scores in Chinese, mathematics and English were selected as the proxy variables of cognitive ability.

Explanatory variables: internet entertainment. This paper referred to two items in the questionnaire to measure children’s internet entertainment. The first item was “whether children use the internet for entertainment”, and it was measured with a dummy variable, which reflects the children’s overall online situation. The second was “the weekly period of internet entertainment”, a categorical variable which reflects different periods of children’s internet entertainment within a week, and it represents non-internet entertainment as 0, internet entertainment only on weekends as 1 and internet entertainment on both weekdays and weekends as 2.

The main variables and explanations selected in this paper are shown in Appendix A. In the analysis process of this paper, we also controlled the factors of children’s demographic characteristics, non-cognitive characteristics, school characteristics and family environment demographic characteristics. In the analysis process of this paper, the factors of family environment were comprehensively considered in relation to four aspects: (1) Family network, which refers to the environment in which children use the internet at home, including whether there is internet at home, parents’ internet habits and parents’ internet supervision; (2) Family education, which refers to the family cultural capital and the educational atmosphere created for children, including parents’ educational background, the number of books at home and parents’ reading habits; (3) Family relationship, which reflects parents’ care for children and family harmony, which is mainly measured by parental company and the parental relationship; (4) Family economic status, which is the basis for parents to provide material conditions and create a growing environment for children and which is measured by two variables, relative family income and residential area, respectively.

At the same time, this paper also controlled for other factors affecting children’s internet entertainment and cognitive ability as much as possible, including children’s gender, age, ethnicity, household registration, school grade, health status, whether they are part of a single-child family and other demographic characteristics, as well as non-cognitive characteristics such as openness, conscientiousness and agreeableness. In addition, school characteristics, such as school type and school ranking, were controlled, and district and county fixed effects were controlled in the model estimation.

### 3.2. Model

In order to explore the net effect of internet entertainment on children’s cognitive ability, on the basis of previous research literature on cognitive ability [30,31], this paper expanded existing research practices to build the ordinary least squares (OLS) model as follows:

(1) $$\;Cognitive_{i}=\alpha +\beta IU_{i}+\gamma Family_{i}+\delta X_{i}+\mu_{i}+\varepsilon_{i\;}\;$$

The software used in the analysis was Stata 14. Equation (1) analyzes the effect of internet entertainment on children’s cognitive ability. The explained variable *Cognitive* refers to children’s cognitive ability, and the core explanatory variable *IU*_i_ is the binary variable which refers to whether the internet is being used for entertainment, and the coefficient refers to the marginal effect of internet entertainment. In terms of control variables, *family* stands for family environment variables (family network, family education, family relations, family economy, etc.). *X_i_* represents children’s demographic characteristics (gender, age, health and whether he/she is the only child in the family), non-cognitive abilities (openness, conscientiousness and agreeableness) and school characteristics (public schools, school quality). *μ_i_* represents the region fixed effect, and *ε_i_* represents the random disturbance term.

Equation (2) explores the effects of internet entertainment in different periods on children’s cognitive ability. Generally speaking, children in junior high school mainly study in class from Monday to Friday and have relatively little time for extra-curricular activities, while they have more free and abundant extra-curricular time on weekends. Therefore, the influence of internet entertainment at different times within a week on cognitive ability may be different. In order to further distinguish the effects of different internet access periods, this paper built the econometric model as follows:

(2) $$\;Cognitive_{i}=\alpha +\beta_{1}Weekend_{i}+\beta_{2}Allweek_{i}+\gamma Family_{i}+\delta X_{i}+\mu_{i}+\varepsilon_{i}\;\;$$

Equation (2) introduces virtual variables of internet entertainment periods into the benchmark model. The variable *Weekend* means using the internet for entertainment only on Saturday and Sunday. *All week* means using the internet for entertainment throughout the whole week. The coefficients 1 and 2 respectively represent the effects of internet entertainment on weekends and on all weekdays. In the empirical analysis, this paper also conducted sample regression according to the family characteristics to test the heterogeneity of the effects of children’s internet entertainment in different family environments.

Equations (3) and (4) explore the influence mechanisms between internet entertainment and children’s cognitive ability. Equation (3) replaces the internet entertainment time (*IUtime_i_)* variable in the benchmark model, and this paper differentiated the impact of weekly online time and daily online time. In addition, combined with the hypothesis regarding cognitive effort, arousal and stimulation theory, it is known that internet entertainment may affect children’s cognitive ability by influencing their cognitive effort, continuous learning and life attitude. In fact, compared with children who use the internet all week, those who use the internet only on the weekend have better control over the internet time, and the positive effect of the internet is more obvious. Therefore, it was necessary to distinguish the heterogeneity of internet time in the analysis. Equation (4) adds the *Weekend* variable and takes the proxy variable (*Y_i_)* as the explained variable for regression, which describes the three dimensions of cognitive effort, continuous learning and life attitude. Among them, *β*_2_ represents the influence of using the internet (only on weekends) on cognitive effort and other variables. In contrast, *β*_1_ represents the influence of other internet entertainment after controlling for using the internet only on weekends.

(3) $$\;\;\;\;Cognitive_{i}=\alpha +\beta IUtime_{i}+\gamma Family_{i}+\delta X_{i}+\mu_{i}+\varepsilon_{i}\;$$

(4) $$Y_{i}=\alpha +\beta_{1}IU_{i}+\beta_{2}Weekend_{i}+\gamma Family_{i}+\delta X_{i}+\mu_{i}+\varepsilon_{i}$$

## 4. Results

### 4.1. Descriptive Statistics

The research samples in this paper were mainly children aged from 13 to 15 in junior middle school. After eliminating the samples with missing key variables, the final number of samples was 10,934. Among them, 7447 children used the internet for entertainment activities. From the perspective of grade distribution, the proportions of students aged 13 and 15 were close, accounting for 50.4% and 49.6%, respectively. From the perspective of gender distribution, the number of female students was slightly larger than that of male students; the proportion of female students was 51.2%, and the proportion of male students was 48.8%.

Table 1 reports the main characteristic variables and describes the statistical results of the full sample, the online sample and the offline sample. From the perspective of cognitive ability, the cognitive ability score of children who use the internet for entertainment was significantly higher than that of children who do not use the internet for entertainment. From the perspective of family characteristics, children having internet access in their families had higher internet penetration rates in richer economic conditions, and more were urban residents. Their parents had a higher education background, lived with more children and often surfed the internet and read books after work. In families with children who do not use the internet, parents had better relationships, and parents monitored their children’s internet usage more strictly. From the perspective of individual characteristics of children, compared with those who do not use the internet, children who use the internet were primarily males, a higher proportion were non-agricultural registered permanent residence and were the only child in a family and were primarily lower-grade students. They scored more highly in openness and agreeableness in non-cognitive ability but lower in conscientiousness.

### 4.2. Regression Results

According to the above analysis strategy, the estimated results of the effects of internet entertainment on children’s cognitive ability were as reported in Table 2. Equations (1) and (2) presented the effects of internet entertainment on children’s cognitive ability, while Equations (3) and (4) listed the effects of internet entertainment in different periods on children’s cognitive ability. In Equation (1), after controlling for children’s individual characteristics, school characteristics and fixed effects of districts, the coefficient of whether children use the internet for entertainment was 0.028, which was significant at the 10% level, indicating that, after excluding children’s individual differences and other factors, children’s internet entertainment has a significant positive association with their cognitive abilities. In Equation (2), family environment and other factors that may affect children’s cognitive ability were further added, and the coefficient of internet entertainment was reduced to 0.002 and made insignificant, indicating that, after considering family characteristics, whether children use internet for entertainment has no significant association with their cognitive abilities. The results seem counterintuitive. Generally speaking, internet entertainment has a great attraction for minor children, especially junior middle school children who are at the stage of entering high school. Once they start to use online games, it is easy for them to indulge in internet entertainment, thus, occupying a large amount of extra-curricular learning and practice time, which has a negative impact on their intellectual development. In fact, children’s internet entertainment itself has no effect on cognitive abilities. There are two possible reasons for this result. First, existing studies did not fully consider the role of the family environment. In addition to the cultural capital, relationship atmosphere and economic status in the family, the internet environment, parents’ supervision of internet entertainment, guidance and demonstration effect also have a substantial impact on children’s internet entertainment. Based on the control variable after the series of children’s personal and family characteristics, we obtained the net effect of internet entertainment. Second, children’s internet entertainment time rather than the internet entertainment itself may have a greater impact. For example, some children have good internet habits, using the internet for entertainment on weekends; however, some other children have poor self-control ability, and they all have the habit of surfing the internet for entertainment no matter whether it is a weekday or a weekend. Because of the above differences in internet situations, internet entertainment has no significant impact on children’s cognitive abilities.

In further analysis, Equations (3) and (4) reported the influences of internet entertainment in different periods on children’s cognitive ability. After adding the fixed-effects model for counties, and controlling the individual features, school and family environment variables, the results showed that the coefficients of using the internet for entertainment on weekends and on all weekdays were 0.059 and −0.048, respectively, which were significant at 1% and 5% levels. The results show that, after considering a series of control variables, surfing the internet for entertainment with no distinction between weekdays and weekends significantly reduces children’s cognitive ability, while surfing the internet for entertainment only on the weekends increases children’s cognitive ability. The above conclusion means that the effects of internet entertainment on children’s cognitive ability at different times are not same, and the actual effect cannot be obtained only by judging whether children use the internet for entertainment. Therefore, it is necessary to distinguish different internet situations.

Equation (4) showed the results of control variables. From the perspective of individual characteristics, the coefficients of gender, grade and health status were significantly positive, while the coefficients of age were significantly negative. The results showed that the cognitive ability of male students was higher than that of female students, the cognitive ability of normal BMI students was higher than that of lean or fat students, the cognitive ability of children in grade 9 was higher than that of children in grade 7 and the cognitive ability of older children was relatively low. As for non-cognitive abilities, the coefficients of openness, conscientiousness and agreeableness were significantly positive, indicating that the non-cognitive abilities were higher. In other words, children with personality characteristics such as curiosity and exploration, serious effort towards study and friendly relationships with classmates also show higher cognitive abilities. As for family education, parents’ education level, the number of books at home and parents’ reading habits were significantly positive, suggesting that good family education resources, especially parents’ education level being higher, the number of books at home and parents’ reading habits, such as reading books and newspapers, promote the development of children’s cognitive ability. As for the family relationship, the influence coefficient of parental company was significantly positive, indicating that children living with parents have a relatively high cognitive ability. As for the family economy, the residential area was significantly positive, indicating that, compared with children living in small towns and rural areas, children who study and live in cities show higher cognitive abilities.

The base model regarded children as a homogeneous group and obtained the average effect of internet entertainment on cognitive ability. However, due to different family environments, the influences of internet entertainment on their cognitive ability are also heterogeneous. In Table 3, Panel A refers to the heterogeneous effect in different family internet environments. Equations (1)–(3) conducted sample regression respectively according to whether the family has internet access, whether the parents have internet habits and whether parents’ internet supervision is strict. The results show that surfing the internet for entertainment only on weekends has a significant association with children’s cognitive ability in different family internet environments. However, the negative influences on children with the internet at home, parents with no internet habits and internet supervision are more evident if they use the internet every day of the week. However, the negative influences are not significant if there is no internet at home, parents without internet habits or if internet supervision is strict.

This is because children spend more time on the internet on weekdays and weekends if there is internet at home, and it occupies a large amount of time otherwise used for extra-curricular learning, reading and sports, which has a more significant, negative impact on their cognitive ability. If parents have regular internet habits, it has a demonstration effect on children’s internet behavior and can correctly guide children to choose beneficial internet entertainment content, reducing the negative influence brought by internet entertainment. If parents have strict supervision of the internet usage, they can effectively supervise children to arrange their online entertainment time reasonably, which is conducive to the formation of good internet habits. However, children with less strict supervision are more likely to indulge in internet entertainment due to a lack of self-control, which has a negative impact on their cognitive ability.

Panel B refers to the heterogeneous effect of different family characteristics. Equations (1)–(3) group regression was conducted according to the educational background of parents, parental relationship and residential area. It should be noted that, for the parent’s educational background, this paper regarded families where both or at least one of the parents have a college degree as high-educational-background families and regarded the rest as low-educational-background families. The results show that, among families with high educational backgrounds, well-connected parents and urban residents, the positive effect of children’s internet entertainment only on weekends is greater. In families with low educational backgrounds, a poor relationship between parents and those living in rural areas, the negative influence brought by children’s internet entertainment on weekends is more prominent. It is worth noting that surfing the internet for entertainment on weekends also had a negative association with children whose parents do not have a good relationship. The above conclusions indicate that the relationships between internet entertainment and children’s cognitive ability in different family environments are obviously heterogeneous.

The quantile regression results of the effect of internet entertainment on children’s cognitive ability are reported in Table 4. In general, the coefficient of surfing the internet for entertainment only on weekends was negative, while the coefficient of surfing the internet all week was positive, which is consistent with the previous conclusions. Meanwhile, the influences of internet entertainment at different times on children’s cognitive ability were heterogeneous. Surfing the internet only on the weekends had a significant, positive association with children whose cognitive ability is below 25% and above 65%, while it had a significant, negative association with children whose cognitive ability is between 25% and 65%. This suggests that internet entertainment on weekends is more conducive to promoting the cognitive development of children with low or medium intelligence levels, while internet entertainment all week actually reduces the cognitive development of children with medium and low intelligence levels.

Based on the theory of self-determination, it can be seen that self-determination is an individual’s free choice of action based on the combination of internal and external motivations and a full understanding of his/her needs and environmental information. For children with low intelligence, arranging their internet entertainment time at the weekend means reducing the obstacles to online learning activities on weekdays, which reflects self-discipline and the parents’ strict controls on internet entertainment. At the same time, it is beneficial for children to obtain information on the outside world through internet entertainment, thus, broadening their visions and improving cognitive ability. Children with higher intelligence usually have a good foundation of cognitive ability. Surfing the internet for entertainment only on weekends means that they can still arrange their extra-curricular time reasonably. Internet entertainment can help to relax learning pressure and improve learning efficiency. For children with medium and low intelligence, surfing the internet for entertainment on weekdays and on weekends means poor self-control. It occupies more time otherwise used for extra-curricular learning and sports, thus, leading to a further decline in their cognitive ability.

### 4.3. Robustness Tests

#### 4.3.1. Conducting the Robustness Test Based on Academic Performance

Academic performance is considered an important indicator to predict children’s cognitive ability. To test whether the influence of internet entertainment on cognitive ability is robust, Table 5 uses academic performance as a proxy variable to conduct the robustness test. In Equation (1), the students’ self-rated scores, measured by the five-level scale, were taken as the explained variables; in Equations (2)–(4), the standardized scores of Chinese, math and English at mid-term examination were taken as the explained variables, respectively. The regression results showed that the coefficient of internet entertainment only on weekends was significantly positive, while the coefficient of internet entertainment on weekdays and weekends was significantly negative, which is consistent with the previous conclusion. These results show that surfing the internet for entertainment on weekends can not only improve children’s cognitive ability compared with children who do not use the internet for entertainment, but can also positively enhance academic performance, and an appropriate level of internet entertainment can help children to alleviate study pressure and improve learning efficiency. On the other hand, surfing the internet for entertainment all week hinders children’s cognitive development and has a negative impact on their academic performance. In other words, these children are addicted to internet games. It leads to declining cognitive ability and academic performance, which is extremely harmful to children’s long-term development.

#### 4.3.2. Using Panel Data to Conduct Robustness Tests

Table 6 shows the robustness test results, which used the matching data in two periods. In Models (1) and (2), OLS estimation was performed using matched sample data in the base period and the later period, respectively. In Models (3) and (4), the fixed year effect was controlled, and the mixed section model and panel data model were estimated by using two-period data. In Model (5), the inter-temporal effect of internet entertainment was obtained by taking internet entertainment in the basic period as the explanatory variable and cognitive ability in the later period as the explained variable. The above results show that, although the coefficient of internet entertainment only on weekends and on both weekdays and weekends were different, surfing the internet for entertainment on weekends promotes children’s cognitive development compared with children who do not use it for entertainment, and surfing the internet for entertainment on weekdays and weekdays reduces children’s cognitive ability, which confirms the above conclusion with robustness.

### 4.4. Influence Mechanism Analysis

According to the above analysis strategy, the influence mechanism of internet entertainment on cognitive ability was further tested, as shown in Table 7. According to the cognitive effort hypothesis, easy access to information through the internet may lead to children’s cognitive inertia and, thus, reduce their effort level. In order to test the above hypothesis, Panel A reflects children’s cognitive effort attitude through students’ responses to questions in the questionnaires, such as “what education degree do you want to achieve” and “Whether the courses of math, Chinese and English are very helpful for my future”. The results show that the influence coefficients of internet entertainment during weekends on higher education expectation and on whether mathematics and Chinese are considered helpful were significantly positive. However, after controlling for internet entertainment during weekends, the influence coefficients of other internet entertainment on higher education expectation and on whether math is considered helpful and whether English is considered helpful were significantly negative. This shows that children who only use the internet for entertainment on weekends hope to obtain higher academic qualifications, believe that learning math and Chinese is beneficial for their future and show a positive cognitive effort attitude and strong learning motivation. However, children who use the internet for entertainment on both weekdays and weekends do not have a strong desire to obtain higher qualifications and hold a negative attitude towards the importance of learning math and English. In other words, internet entertainment reduces the cognitive effort attitude of these groups and, thus, has a negative impact on their cognitive ability.

According to the arousal hypothesis, internet entertainment is characterized by high arousal and rapid presentation, and prolonged internet entertainment interferes with children’s attention, concentration and learning duration, thus, affecting their attitude towards learning and life and causing bad behaviors. Panel B reflects the children’s continuous learning behavior through their responses to two questions in the questionnaire, “I often attend classes” and “I am often late for classes”, and reflects their life attitude through their responses to questions such as “feeling life is boring and sad”. The results showed that children who use the internet for entertainment only on weekends are less likely to be late for class, believe that their lives are meaningful and have fewer negative emotions such as sadness. However, children with other internet entertainment conditions are more likely to be late for class, skip class and have other unfavorable behaviors and are more likely to have negative emotions such as a sense of meaninglessness in life and sadness. It can be seen that moderate internet entertainment on weekends does not induce children’s bad behavior but increases the fun of life and avoids the occurrence of negative emotions. However, excessive internet entertainment on both weekdays and weekends is not conducive to the formation of children’s continuous learning habits and has a negative impact on their attitude towards life, thus, hindering the development of children’s cognitive ability.

### 4.5. Additional Analysis

The above analysis shows that whether or not children use the internet for entertainment has no obvious effect on their cognitive ability. However, from the perspective of internet entertainment in different periods, surfing the internet on both weekdays and weekends reduces their cognitive ability, while surfing the internet only on weekends promotes their cognitive ability development. Why do different periods for internet entertainment lead to different results? This paper tries to explain its influence mechanism by combining relevant theories.

**Figure 1:**
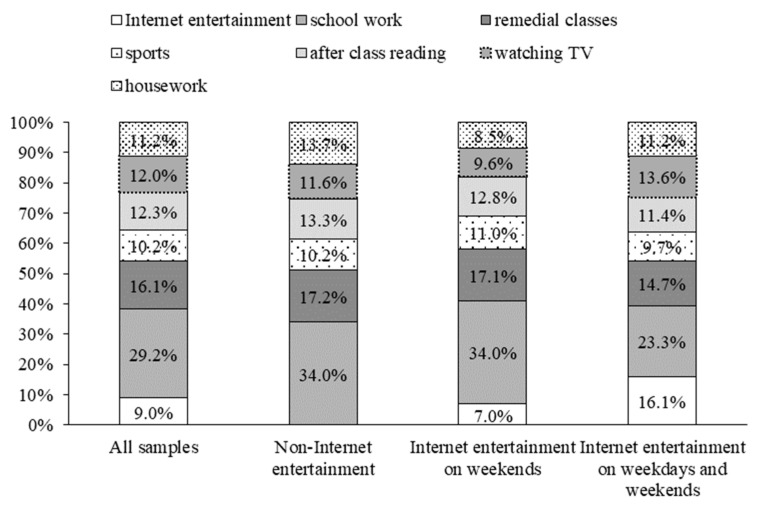
Children’s weekly extra-curricular time allocation.

Figure 1 shows children’s weekly extra-curricular time allocation. The figure shows differences in the distribution of extra-curricular time among teenagers who use the internet for different periods. Overall, children spend the most time on schoolwork, remedial classes and after-class reading, which accounted for 29.2%, 16.1% and 12.3%, respectively, while internet entertainment accounted for 9.0%. Teenagers who do not use the internet for entertainment spend more than average time on schoolwork, remedial classes and extra-curricular reading, and they also spend more time on housework. Combined with the above analysis, it can be seen that the family economic conditions of the teenagers who have no internet entertainment are poor, most of them live in rural areas and they spend their extra-curricular time learning and doing housework. For children who only use the internet for entertainment on weekends, only 7% of their time is spent on internet entertainment, which is lower than the average. They also spend more time than average on schoolwork, remedial classes and sports and less time than average watching TV and doing housework. For children who use the internet for entertainment on both weekdays and weekends, the time spent on schoolwork, remedial classes, sports and extra-curricular reading is relatively short, but the time spent on internet entertainment and watching TV is significantly longer. It can be seen that children who only use the internet for entertainment on weekends pay more attention to their comprehensive development. They not only have good internet habits but can also better balance the time used to study, read and play sports. Children who use the internet for entertainment every day have poor self-control and are more likely to indulge in entertainment activities such as online games and TV shows, thus, affecting their studying performance and capability development.

The influence of internet entertainment time on cognitive ability is further reported in Table 8. The results showed that the coefficient of average weekly internet entertainment time in Equation (1) was −0.009, which was significantly negative at the 1% level, consistent with the hypothesis of time substitution theory. In Equations (2)~(4), the influences of daily internet entertainment time on children’s cognitive ability during the weekdays and weekends were estimated. The results show that weekday internet entertainment time had a negative association with children’s cognitive ability. However, there was an “inverted U” relationship between children’s internet entertainment time on weekends and their cognitive ability. That is to say, with the increase of internet entertainment time on weekends, children’s cognitive ability increases in the beginning and then decreases. The reason is that an appropriate amount of internet entertainment time on weekends can relax children’s moods and relieve their studying stress. Still, excessive time spent on internet entertainment during the weekdays and weekends occupies the time otherwise spent on other extra-curricular learning activities, which harms children’s physical and mental well-being.

## 5. Findings and Discussion

This study investigated the effect of internet entertainment on children’s cognitive ability and its influencing mechanism. First of all, this paper reviewed the literature on children’s internet entertainment, cognitive ability and family environment and put forward research hypotheses. Based on the data of China Education Panel Survey (CEPS), this paper built an econometric model to empirically test the effect of internet entertainment on children’s cognitive ability, and heterogeneity analysis and robustness tests were conducted. After that, the paper further discussed the influence mechanism of internet entertainment on cognitive ability during different times in the week. The statistical techniques used in the paper were ordinary least squares regression (OLS), descriptive statistics and T-test analysis, and the software used in the empirical analysis was Stata 14. In summary, this research was an observational and empirical study, and it reviewed the previous literature and put forward new research conclusions.

The empirical results verified the following conclusions: First, internet entertainment has no significant impact on children’s cognitive ability, but additional analysis provided preliminary evidence suggesting that internet entertainment can be beneficial to children who use it for entertainment only on weekends but detrimental for those who spend leisure time online daily. The positive effects are more obvious in teenager groups with medium and high cognitive abilities, while the negative effects are more obvious in teenager groups with low and medium cognitive abilities. Second, the effects of internet entertainment on children’s cognitive ability in different family environments are heterogeneous, which is consistent with previous papers [24]. Surfing the internet for entertainment only on weekends has a greater positive impact on children with high education, good parental relationships and urban families, while surfing the internet for entertainment on both weekdays and weekends has a greater negative impact on children who have access to the internet at home and parents with no internet habits and a lack of supervision, and the negative impact is obvious on children with low education, poor parental relationship and rural families. Third, internet entertainment mainly affects children’s cognitive ability by influencing their allocation of extra-curricular time, learning and life attitude. Children who spend more time on internet entertainment during weekdays have decreased cognitive ability, while moderate internet entertainment on weekends can improve their cognitive ability. Moreover, children who use the internet for entertainment on weekends have a positive cognitive effort attitude and strong learning motivation, and they are inclined to think that life is meaningful and have fewer negative emotions such as sadness. As for the research conclusion, because the survey data and research variables will be further updated in the future, subsequent studies could further analyze the positive and negative effects of internet entertainment and discuss its future return on human capital.

The risks and benefits of internet entertainment for children’s cognitive development have long been debated [32]. The conclusion of this paper provides an alternative view, that is, children’s internet entertainment behavior itself does not mean a decrease of cognitive ability, and moderate internet entertainment can promote children’s cognitive ability. In addition, the heterogeneity of the family environment was considered in this study. It was concluded that family is the most important environment that children live in and rely on for growth and development [33]. When discussing the relationship between internet entertainment and children’s cognitive ability, it is of great practical significance to consider the role of a family environment. Due to differences in the family network environment, educational resources and residential areas, internet entertainment may differently affect children’s cognitive ability [23]. Therefore, it is necessary to distinguish the heterogeneity of family characteristics. In recent years, with the increase of children’s internet penetration rate, they use internet entertainment applications starting at a younger age than previously. Most parents hold an unsupportive attitude towards their children’s consumption of internet entertainment in real life [34]. To prevent their children from becoming addicted to the internet, they often prohibit their children from using the internet for entertainment. However, in the digital era, children unfamiliar with the internet may not be able to develop digital competencies, and it has a negative impact on children’s rapid adaptation to digital development.

## 6. Implications and Limitations

Our analysis has some implications for internet entertainment and children’s cognitive development. This paper suggests that families, schools and society should pay more attention to guiding children to develop good internet habits and better control their internet entertainment time, with optimal effects not necessarily being achieved only through strict prohibition. From the perspective of family guidance, parents should help children to cultivate healthy and reasonable entertainment habits, thus, arranging reasonable internet entertainment time [35]. At the same time, parents should face up to the benefits brought by internet entertainment, and they can use internet entertainment as a means to motivate children so as to form a harmonious family internet atmosphere and further promote the development of children’s cognitive abilities. From the view of schools and society, relevant government departments need to strengthen the regulation of the internet entertainment industry, screen inappropriate information in a more timely way, better regulate the relevant internet applications and enforce the real-name registration system [36] and limit children using internet entertainment services which are incompatible with their age. Finally, from the perspective of children themselves, it is necessary to develop a self-disciplined internet habit and take internet entertainment as a means to relax during leisure time and control the time spent on internet entertainment. In addition, it is necessary to maintain a motivated attitude towards learning and life and use internet resources to acquire new knowledge and information.

There are some issues that still need to be further discussed. From the perspective of research objects, due to data limitations (China Education Panel Survey), this paper focused on children in the Chinese context. In the future, it is suggested that the research objects be further expanded to pay attention to children in the English context or in other countries. From the perspective of the research methods, although this paper used the panel data model to verify the conclusion with robustness, future research could attempt to use instrumental variables, breakpoint regression and the experimental method to analyze internet entertainment effects on children’s cognitive ability. From the perspective of research content, this paper focused on cognitive ability, and future research could try to comprehensively study non-cognitive ability, physical and mental health, sleep quality, academic performance and other variables. Because the database only contained information about overall internet entertainment, this paper discussed the impact of children’s overall internet entertainment. However, there are different manners of internet entertainment, such as internet games, online music, internet news, online social networking, online video, etc. Future studies could further focus on children’s different manners of internet entertainment and analyze the impact of specific internet entertainment on children’s cognitive abilities, and research could also be conducted on the impact of internet applications on left-behind children or children in remote areas and other special groups.

## Figures and Tables

**Table 1:** Descriptive statistics.

Variables	All Samples		Internet Entertainment		Non-Internet Entertainment		*T*-Test
	Mean	S.D.	Mean	S.D.	Mean	S.D.	
Cognitive score	0.124	0.837	0.160	0.836	0.045	0.832	−0.115 ***
Internet at home	0.663	0.473	0.792	0.406	0.387	0.487	−0.405 ***
Parents’ internet habit	0.624	0.484	0.704	0.457	0.456	0.498	−0.248 ***
Parents’ internet supervision	0.652	0.476	0.597	0.491	0.769	0.422	0.171 ***
Father’s education	10.65	3.152	10.89	3.126	10.14	3.146	−0.753 ***
Mother’s education	9.948	3.495	10.29	3.375	9.225	3.635	−1.061 ***
Number of books at home	3.304	1.178	3.398	1.135	3.102	1.241	−0.295 ***
Parent’s reading habits	0.749	0.434	0.783	0.413	0.677	0.468	−0.106 ***
Parental company	0.871	0.335	0.881	0.324	0.851	0.357	−0.030 ***
Parental relationship	0.85	0.357	0.844	0.363	0.863	0.344	0.019 **
Family income	3.029	0.522	3.088	0.493	2.902	0.557	−0.186 ***
Residential area	0.564	0.496	0.617	0.486	0.450	0.498	−0.167 ***
Gender	0.488	0.500	0.512	0.500	0.434	0.496	−0.078 ***
Household registration	0.479	0.500	0.518	0.500	0.395	0.489	−0.123 ***
Single-child family	0.479	0.500	0.513	0.500	0.407	0.491	−0.107 ***
School grade	0.496	0.500	0.486	0.500	0.519	0.500	0.033 ***
Openness	3.195	0.596	3.211	0.599	3.163	0.589	−0.047 ***
Conscientiousness	3.345	0.655	3.307	0.665	3.425	0.626	0.117 ***
Agreeableness	3.185	0.681	3.193	0.674	3.167	0.695	−0.026 *
No. of observations	10,934		7447		3487		——

Note: The superscripts ***, ** and * indicate significance at the 1%, 5% and 10% confidence levels, respectively.

**Table 2:** Regression results.

Variables	(1)	(2)	(3)	(4)
Internet entertainment	0.028 * (0.016)	0.002 (0.017)		
Internet entertainment on weekends			0.089 *** (0.019)	0.059 *** (0.02)
Internet entertainment on weekdays and weekends			−0.024 (0.018)	−0.048 ** (0.019)
Gender	0.017 (0.015)	0.028 * (0.015)	0.014 (0.015)	0.025 * (0.015)
Age	−0.142 *** (0.011)	−0.122 *** (0.011)	−0.140 *** (0.011)	−0.120 *** (0.011)
Ethnic group	0.014 (0.035)	0.015 (0.035)	0.014 (0.035)	0.015 (0.035)
Household registration	0.088 *** (0.018)	0.017 (0.019)	0.085 *** (0.018)	0.015 (0.019)
Single-child family	0.046 ** (0.018)	0.006 (0.018)	0.042 ** (0.018)	0.002 (0.018)
School grade	0.28 *** (0.027)	0.249 *** (0.027)	0.272 *** (0.027)	0.242 *** (0.027)
Health	0.027 * (0.015)	0.029 ** (0.015)	0.026 * (0.015)	0.028 * (0.015)
Openness	0.099 *** (0.014)	0.079 *** (0.014)	0.100 *** (0.014)	0.080 *** (0.014)
Conscientiousness	0.044 *** (0.013)	0.049 *** (0.013)	0.040 *** (0.013)	0.045 *** (0.013)
Agreeableness	0.067 *** (0.011)	0.052 *** (0.011)	0.065 *** (0.011)	0.051 *** (0.011)
Internet at home		0.031 (0.020)		0.032 (0.02)
Parents’ internet habit		0.018 (0.018)		0.020 (0.018)
Parents’ internet supervision		−0.002 (0.016)		−0.007 (0.015)
Father’s education		0.019 *** (0.003)		0.019 *** (0.003)
Mother’s education		0.006 * (0.003)		0.006 * (0.003)
Number of books at home		0.042 *** (0.008)		0.042 *** (0.008)
Parents’ reading habits		0.043 ** (0.019)		0.041 ** (0.019)
Parental company		0.039 * (0.022)		0.037 * (0.022)
Parental relationship		−0.015 (0.02)		−0.016 (0.02)
Family income		−0.001 (0.016)		−0.0004 (0.015)
Residential area		0.045 ** (0.019)		0.042 ** (0.019)
School characteristics	YES	YES	YES	YES
District fixed effect	YES	YES	YES	YES
Adjusted R^2^	0.198	0.210	0.201	0.212
N	10,934	10,934	10,934	10,934

Note: The superscripts ***, ** and * indicate significance at the 1%, 5% and 10% confidence levels, respectively.

**Table 3:** Heterogeneous analysis results.

Panel A: The Heterogeneous Effect in Different Family Internet Environments						
Variables	(1) Internet at Home		(2) Parents’ Internet Habit		(3) Parents’ Internet Supervision	
	Yes	No	Yes	No	Strict	Relaxed
Internet entertainment on weekends	0.063 ** (0.025)	0.069 ** (0.034)	0.063 ** (0.025)	0.073 ** (0.032)	0.049 ** (0.023)	0.073 ** (0.037)
Internet entertainment on weekdays and weekends	−0.043 * (0.026)	−0.032 (0.031)	−0.033 (0.025)	−0.057 * (0.031)	−0.027 (0.023)	−0.083 ** (0.036)
Adjusted R^2^	0.186	0.161	0.179	0.191	0.197	0.240
N	7246	3688	6828	4106	7127	3807
**Panel B: The Heterogeneous Effect in Different Family Characteristics**						
**Variables**	**(1) Parents’ Education**		**(2) Parents’ Relationship**		**(3) Residential Area**	
	**High**	**Low**	**Good**	**Bad**	**City**	**Town and Rural Area**
Internet entertainment on weekends	0.116 *** (0.041)	0.045 ** (0.022)	0.085 *** (0.021)	−0.101 ** (0.05)	0.071 *** (0.026)	0.063 ** (0.03)
Internet entertainment on weekdays and weekends	−0.006 (0.043)	−0.056 *** (0.022)	−0.031 (0.021)	−0.151 *** (0.049)	−0.037 (0.026)	−0.049 * (0.028)
Adjusted R^2^	0.146	0.190	0.211	0.224	0.201	0.158
N	2400	8534	9295	1639	6167	4767

Note: The superscripts ***, ** and * indicate significance at the 1%, 5% and 10% confidence levels, respectively.

**Table 4:** Quantile regression results.

Variables	QR_10	QR_25	QR_35	QR_50	QR_65	QR_75	QR_90
Internet entertainment on weekends	0.097 *** (0.034)	0.046 * (0.027)	0.023 (0.028)	0.036 (0.027)	0.067 ** (0.029)	0.075 ** (0.031)	0.059 * (0.035)
Internet entertainment on weekdays and weekends	−0.053 (0.037)	−0.064 ** (0.028)	−0.077 *** (0.027)	−0.083 *** (0.026)	−0.050 * (0.027)	−0.041 (0.029)	−0.012 (0.033)
Adjusted R^2^	0.082	0.132	0.136	0.140	0.138	0.129	0.084
N	10,934	10,934	10,934	10,934	10,934	10,934	10,934

Note: The superscripts ***, ** and * indicate significance at the 1%, 5% and 10% confidence levels, respectively.

**Table 5:** Influence of internet entertainment on academic performance.

Variables	(1) Self-Rated Scores	(2) Chinese Scores	(3) Math Scores	(4) English Scores
Internet entertainment on weekends	0.047 * (0.027)	0.477 ** (0.222)	0.751 *** (0.232)	0.490 ** (0.23)
Internet entertainment on weekdays and weekends	−0.136 *** (0.027)	−1.027 *** (0.224)	−1.384 *** (0.235)	−1.078 *** (0.225)
Adjusted R^2^	0.045	0.133	0.056	0.131
N	10,198	10,934	10,934	10,934

Note: The superscripts ***, ** and * indicate significance at the 1%, 5% and 10% confidence levels, respectively.

**Table 6:** Robustness tests of matching data in two periods.

Variables	(1)	(2)	(3)	(4)	(5)
	Matched Sample in Base Period	Matched Sample in Later Period	Mixed Section Data in Two Periods	Panel Data in Two Periods	Inter-Temporal Effect
Internet entertainment on weekends	0.083 *** (0.031)	0.132 *** (0.031)	0.104 *** (0.022)	0.073 *** (0.022)	0.113 *** (0.026)
Internet entertainment on weekdays and weekends	−0.050 * (0.03)	−0.059 ** (0.029)	−0.058 *** (0.021)	−0.036 * (0.02)	−0.098 *** (0.026)
Year control	NO	NO	YES	YES	NO
Adjusted R^2^	4783	4783	9566	9566	4783
N	0.190	0.238	0.229	0.2312	0.240

Note: The superscripts ***, ** and * indicate significance at the 1%, 5% and 10% confidence levels, respectively.

**Table 7:** Influence mechanism of internet entertainment on children’s cognitive ability.

Panel A: Cognitive effort				
Variables	(1) Higher Education Expectation	(2) Mathematics Is Helpful	(3) Chinese Is Helpful	(4) English Is Helpful
Internet entertainment	−0.294 *** (0.038)	−0.074 *** (0.020)	−0.021 (0.019)	−0.046 ** (0.021)
Internet entertainment on weekends	0.189 *** (0.034)	0.084 *** (0.019)	0.039 ** (0.017)	0.012 (0.019)
Adjusted R^2^	0.185	0.139	0.106	0.157
N	10,603	10,926	10,926	10,914
**Panel B: Continuous learning and life attitude**				
**Variables**	**(1) Always Late** **for Class**	**(2) Always Skip Class**	**(3) Feeling Life** **Is Boring**	**(4) Feeling Life** **Is Sad**
Internet entertainment	0.080 *** (0.015)	0.018 * (0.010)	0.087 *** (0.026)	0.045 * (0.026)
Internet entertainment on weekends	−0.046 *** (0.014)	−0.012 (0.008)	−0.061 ** (0.024)	−0.066 *** (0.023)
Adjusted R^2^	0.052	0.020	0.084	0.075
N	10,929	10,925	10,934	10,934

Note: The superscripts ***, ** and * indicate significance at the 1%, 5% and 10% confidence levels, respectively.

**Table 8:** Influence of internet entertainment time on cognitive ability.

Variables	(1)	(2)	(3)	(4)
Average weekly internet entertainment time	−0.009 *** (0.001)			
Daily internet entertainment time during weekdays		−0.061 *** (0.006)	−0.061 *** (0.007)	−0.060 *** (0.007)
Daily internet entertainment time on weekends			0.0003 (0.005)	0.028 ** (0.011)
The square of daily internet entertainment time on weekends/100				−0.444 *** (0.162)
Adjusted R2	0.216	0.217	0.217	0.218
N	10,934	10,934	10,934	10,934

Note: The superscripts *** and ** indicate significance at the 1% and 5% confidence levels, respectively.

## Data Availability

The authors will share data from the study upon reasonable request to the corresponding author.

## Appendix A

**Table A1:** Variables description.

Dimension	Variable	Explanation
Internet entertainment	Whether children use internet for entertainment	Yes = 1, No = 0
	The weekly period of internet entertainment	Non-internet entertainment = 0, Only on weekends = 1, On both weekdays and weekends = 2
	Internet entertainment time	Average daily internet entertainment time during weekdays or weekends (unit: hours)
Cognitive ability	Cognitive score	Standardized scores on cognitive tests (using the 3PL model)
Non-cognitive ability	Openness	React quickly, be able to learn new knowledge quickly and be curious about new things (four-level scale)
	Conscientiousness	Keep going to school and be able to do homework to the best of their ability (four scales)
	Agreeableness	Be close and friendly to classmates or people around (four-level scale)
Family network	Internet at home	Whether there is internet at home? (Yes = 1, No = 0)
	Parents’ internet habits	Whether parents have internet habits after work? (Yes = 1, No = 0)
	Parents’ internet supervision	Are parents very strict about children’s internet time? (Yes = 1, No = 0)
Family education	Father’s education	Years of father’s education background (unit: years)
	Mother’s education	Years of mother’s education background (unit: years)
	Number of books at home	Few = 1, Relatively few = 2, Generally = 3, Relatively many = 4, Many = 5
	Parent’s reading habits	Whether parents have the reading habit after work? (Yes = 1, No = 0)
Family relationship	Parental company	Whether the children live with their parents? (Yes = 1, No = 0)
	Parental relationship	Whether parents maintain a good relationship? (Yes = 1, No = 0)
Family economic status	Family income	Very poor = 1, Relatively poor = 2, Medium = 3, Relatively rich = 4, Very rich = 5
	Residential area	Central and marginal urban areas = 1, Towns and villages = 0
Demographic characteristic	Gender	Male = 1, Female = 0
	Age	Actual age by year of survey
	Nationality	The Han nationality = 1, Others = 0
	Household registration	Non-agricultural registration = 1, Agricultural registration = 0
	Single child family	Yes = 1, No = 0
	School grade	Grade 9 = 1, Grade 7 = 0
	Health	Is your BMI normal? (Normal = 1, Underweight or overweight = 0)
School characteristics	School type	Whether it is a public school? (Yes = 1, Private and others = 0)

## References

[1] Barrow L., Markman L., Rouse C.E. (2009). Technology’s edge: The educational benefits of computer-aided instruction. Am. Econ. J..

[2] Malamud O., Pop-Eleches C. (2011). Home computer use and the development of human capital. Q. J. Econ..

[3] Algan Y., Fortin N.M. (2018). Computer gaming and the gender math gap: Cross-country evidence among teenagers: Work, occupation, earnings and retirement. Transitions through the Labor Market.

[4] Kuss D.J., Lopez-Fernandez O. (2016). Internet addiction and problematic Internet use: A systematic review of clinical research. World J. Psychiatry.

[5] Ye J., Zhang M. (2019). Where Will Left-behind Children be Taken by the Game?. China Youth Daily.

[6] Zheng L., Weng Q., Gong X. (2021). Does preschool attendance affect the urban-rural cognition gap among middle school students? Evidence from China Education Panel Survey. J. Chin. Sociol..

[7] Fang C., Wang G., Huang B. (2019). Can information technology promote the development of students’ cognitive ability? Net effect estimation based on CEPS. Open Educ. Res..

[8] Akee R., Copeland W., Costello E.J., Simeonova E. (2018). How does household income affect child personality traits and behaviors?. Am. Econ. Rev..

[9] Fang G., Hou Y. (2019). How family socioeconomic status affects the cognitive development of junior high school students. Glob. Educ. Outlook.

[10] Donoso G., Casas F., Rubio A., Céspedes C. (2021). Mediation of problematic use in the relationship between types of internet use and subjective well-being in schoolchildren. Front. Psychol..

[11] Jackson L.A., Von Eye A., Biocca F.A., Barbatsis G., Zhao Y., Fitzgerald H.E. (2006). Does home internet use influence the academic performance of low-income children?. Dev. Psychol..

[12] Zheng L., Qi X., Zhu Z., Zhang D.Q. (2021). Family Internet access and the cognitive gap between urban and rural junior high school students. Educ. Dev. Res..

[13] Wang J.N., Zhang Z. (2019). The influence of mobile Internet new media on the cognition and behavior of the second generation left behind children. New Media Res..

[14] Kalenkoski C.M., Pabilonia S.W. (2012). Time to work or time to play: The effect of student employment on homework, sleep, and screen time. Labour Econ..

[15] Heckman J.J. (2007). The economics, technology, and neuroscience of human capability formation. Proc. Natl. Acad. Sci. USA.

[16] Vigdor J.L., Ladd H.F., Martinez E. (2014). Scaling the digital divide: Home computer technology and student achievement. Econ. Inq..

[17] Ophir E., Nass C., Wagner A.D. (2009). Cognitive control in media multitaskers. Proc. Natl. Acad. Sci. USA.

[18] Beuermann D.W., Cristia J., Cueto S., Malamud O., Cruz-Aguayo Y. (2015). One laptop per child at home: Short-term impacts from a randomized experiment in Peru. Am. Econ. J. Appl. Econ..

[19] Walsh J.L., Fielder R.L., Carey K.B., Carey M.P. (2013). Female college students’ media use and academic outcomes: Results from a longitudinal cohort study. Emerg. Adulthood.

[20] Gentile D.A. (2011). The multiple dimensions of video game effects. Child Dev. Perspect..

[21] Cardoso-Leite P., Green C.S., Bavelier D. (2015). On the impact of new technologies on multitasking. Dev. Rev..

[22] Bulman G., Fairlie R.W. (2016). Technology and education: Computers, software, and the Internet. Handbook of the Economics of Education.

[23] Vaala S.E., Bleakley A. (2015). Monitoring, mediating, and modeling: Parental influence on adolescent computer and Internet use in the United States. J. Child. Media.

[24] Zou H., Jin S., Wu S. (2014). The relationship between family economic status and Internet addiction among adolescents: The moderating effect of interpersonal relationship. Educ. Res. Exp..

[25] Deng L., Fang X., Wu M., Zhang J., Liu Q. (2013). Family Environment, Parent-Child Attachment and Adolescent Internet Addiction. Psychol. Dev. Educ..

[26] Mathiesen K. (2013). The Internet, children, and privacy: The case against parental monitoring. Ethics Inf. Technol..

[27] Phillipson S., Phillipson S.N. (2007). Academic expectations, belief of ability, and involvement by parents as predictors of child achievement: A cross-cultural comparison. Educ. Psychol..

[28] Yang C. (2006). Social class difference in education expectation: The relationship between social status and parental education expectation. Tsinghua Univ. Educ. Res..

[29] Biagi F., Loi M. (2013). Measuring ICT use and learning outcomes: Evidence from recent econometric studies. Eur. J. Educ..

[30] Frey M.C., Detterman D.K. (2004). Scholastic assessment or g? the relationship between the scholastic assessment test and general cognitive ability. Psychol. Sci..

[31] LePine J.A. (2003). Team adaptation and postchange performance: Effects of team composition in terms of members’ cognitive ability and personality. J. Appl. Psychol..

[32] Danovitch J.H. (2019). Growing up with Google: How children’s understanding and use of internet-based devices relates to cognitive development. Human Behav. Emer. Tech..

[33] Liang W.Y., Xiao-Mei Y.E., Tao L.I. (2018). How does parental involvement affect the cognitive ability of migrant children:an empirical study based on CEPS database. J. Educ. Stud..

[34] Billari F.C., Giuntella O., Stella L. (2018). Broadband internet, digital temptations, and sleep. J. Econ. Behav. Organ..

[35] Jiang S., Dong L. (2020). Association between deprivation and cognitive ability among Chinese adolescents: Examining the mechanisms of parental involvement in a rural–urban dual system. Curr. Psychol..

[36] Deci E.L., Ryan R.M., Williams G.C. (1996). Need satisfaction and the self-regulation of learning. Learn. Individ. Differ..

